# Dose-dependent effect of human milk on Bronchopulmonary dysplasia in very low birth weight infants

**DOI:** 10.1186/s12887-020-02394-1

**Published:** 2020-11-16

**Authors:** Yan Xu, Zhangbin Yu, Qianqian Li, Jinjun Zhou, Xiaoguang Yin, Yuelan Ma, Yujie Yin, Shanyu Jiang, Rongping Zhu, Yue Wu, Liangrong Han, Yan Gao, Mei Xue, Yu Qiao, Lingling Zhu, Wenjuan Tu, Mingfu Wu, Jun Wan, Weiyuan Wang, Xiaoyi Deng, Shuangshuang Li, Sannan Wang, Xiaoqing Chen, Qin Zhou, Jinxiu Wang, Rui Cheng, Jun Wang, Shuping Han

**Affiliations:** 1 Department of Neonatology, The Affiliated Hospital of Xuzhou Medical University, No. 99, Huaihai West Road, Xuzhou, 221000 Jiangsu Province China; 2 Department of Neonatology, The Women’s Hospital of Nanjing Medical University, Nanjing Maternity and Child Health Care Hospital, Nanjing, 210004 Jiangsu China; 3 Department of Neonatology, Xuzhou Maternity and Child Health Care Hospital, Xuzhou, 210009 Jiangsu China; 4 Department of Neonatology, Nantong Maternity and Child Health Care Hospital, Nantong, 226001 Jiangsu China; 5 Department of Neonatology, Anhui women and Child Health Care Hospital, Hefei, 230001 Anhui China; 6 Department of Neonatology, The Affiliated Suzhou Hospital of Nanjing Medical University and Suzhou Municipal Hospital, Suzhou, 215002 Jiangsu China; 7 Department of Neonatology, Jiangsu Women and Children Health Hospital, Nanjing, 210036 Jiangsu China; 8 Department of Neonatology, Wuxi Maternity and Child Health Care Hospital, Wuxi, 214002 Jiangsu China; 9 Department of Neonatology, Changzhou Maternity and Child Health Care Hospital, Changzhou, 213003 Jiangsu China; 10 Department of Neonatology, Nanjing Children’s Hospital, Nanjing, 210008 Jiangsu China; 11 Department of Neonatology, Huaian Maternity and Child Health Care Hospital, Huaian, 223002 Jiangsu China; 12 Department of Neonatology, Lianyungang Maternity and Child Health Care Hospital, Lianyungang, 222000 Jiangsu China; 13 Department of Neonatology, Taizhou People’s Hospital, Taizhou, 225300 Jiangsu China; 14 Department of Neonatology, Affiliated Hospital of Jiangsu University, Zhenjiang, 212001 Jiangsu China; 15 Department of Neonatology, Northern Jiangsu People’s Hospital, Yangzhou, 225001 Jiangsu China; 16 Department of Neonatology, Changzhou Children’s Hospital, Changzhou, 213003 Jiangsu China; 17 Department of Neonatology, Affiliated Hospital of Yangzhou University, Yangzhou, 225001 Jiangsu China; 18 Department of Neonatology, Jiangyin People’s Hospital, Jiangyin, 214400 Jiangsu China; 19 Department of Neonatology, Suqian Maternity Hospital, Suqian, 223800 Jiangsu China; 20 Department of Pediatrics, Women’s Hospital of Nanjing Medical University, Nanjing Maternity and Child Health Care Hospital, No. 123 Tian Fei Xiang, Mo Chou Road, Nanjing, Jiangsu Province 210004 China

**Keywords:** Very low birth weight, Bronchopulmonary dysplasia, Human milk, Complications

## Abstract

**Background and aim:**

Human milk has potential protective effects against bronchopulmonary dysplasia (BPD). However, studies on the association between the dose of human milk and BPD in China are limited. This study aimed to evaluate the dose-dependent effects of human milk on BPD and other neonatal morbidities in very low birth weight (VLBW) infants.

**Methods:**

This retrospective cohort study of preterm infants was conducted on preterm infants of gestational age ≤ 34 weeks and birth weight < 1500 g admitted to the multicenter clinical research database for breastfeeding quality improvement in Jiangsu province. The multivariate analysis was performed to compare the effect outcomes of daily graded doses [1–24 mL/(kg · day), 25–49 mL/(kg · day), and ≥ 50 mL/(kg · day) of body weight] of human milk on neonatal outcomes throughout the first 4 weeks of life versus a reference group receiving no human milk. The models were adjusted for potential confounding variables.

**Results:**

Of 964 included infants, 279 (28.9%) received exclusive preterm formula, 128 (13.3%) received 1–24 ml/(kg · day), 139 (14.4%) received 25–49 ml/(kg · day), and 418 (43.4%) received ≥50 ml/(kg · day) human milk for the first 4 weeks of life. Compared with infants receiving exclusive formula, those receiving the highest volume of human milk daily [≥50 mL/(kg · day)] had lower incidences of BPD [27.5% in ≥50 mL/(kg · day) vs 40.1% in 0 mL/(kg · day) human milk, *P* = 0.001)], moderate and severe BPD [8.9% in ≥50 mL/(kg · day) vs 16.1% in 0 mL/(kg · day), *P* = 0.004], necrotizing enterocolitis [NEC; 3.8% in ≥50 mL/(kg · day) vs 10.8% in 0 mL/(kg · day), *P* = 0.001], late-onset sepsis [LOS; 9.3% in ≥50 mL/(kg · day) vs 19.7% in 0 mL/(kg · day), *P* <0.01], and extrauterine growth retardation [EUGR; 38.5% in ≥50 mL/(kg · day) vs 57.6% in 0 mL/(kg · day), *P* <0.01)]. The logistic regression indicated that those receiving ≥50 ml/kg · day human milk had lower odds of BPD [adjusted odds ratio (AOR) 0.453; 95% confidence interval (CI): 0.309, 0.666], moderate and severe BPD (AOR 0.430; 95% CI: 0.249, 0.742), NEC (AOR 0.314; 95% CI: 0.162, 0. 607), LOS (AOR 0.420; 95% CI: 0.263, 0.673), and EUGR (AOR 0.685; 95% CI: 0.479, 0.979).

**Conclusions:**

A daily threshold amount of ≥50 ml/(kg · day) human milk in the first 4 weeks of life was associated with lower incidence of BPD as well as NEC, LOS, and EUGR in VLBW infants.

**Trial registration:**

ClinicalTrials.gov Identifier: NCT03453502. Registration date: March 5, 2018. This study was retrospectively registered.

**Supplementary information:**

**Supplementary information** accompanies this paper at 10.1186/s12887-020-02394-1.

## Background

With the growth in survival rates of very low birth weight (VLBW) infants, neonatal complications, such as BPD, NEC, LOS, and EUGR, threaten the life and prognosis of neonates. Especially bronchopulmonary dysplasia (BPD) is an increasingly common adverse respiratory outcome [1]. BPD prolongs neonatal intensive care unit (NICU) hospitalization and impacts long-term pulmonary morbidity and chronic neurologic impairment [2, 3]. BPD is affected by multiple factors, including exposure of the immature lung to hypoxia and inflammation, and inadequate nutrition, among others [4, 5]. Preventive and therapeutic strategies are not clear [6]. Human milk has potent protective mechanisms targeting oxidative stress, inflammation, and inadequate nutrition [7]. However, exclusive breastfeeding rates in the early years of life are very low in China, and most NICUs use mixed feeding [8]. Multicenter studies conducted in China on the association between the dose of human milk in mixed feeding and BPD are lacking. This study was performed to evaluate the dose-dependent impact of human milk received up to the end of week 4 of life on BPD in VLBW infants.

## Methods

### Participating centers

A multicenter coordination group for improving breastfeeding quality, with representation from 19 NICUs in tertiary hospitals was established before data collection. Eighteen NICUs were situated in Jiangsu province, and one in Anhui province. Of the NICUs, 10 were at maternity and child healthcare hospitals, 2 were at children’s hospitals and 7 were at a general hospital.

Breastfeeding was encouraged at all the NICUs, two of which had human milk banks. The Women’s Hospital of Nanjing Medical University was responsible for coordinating the survey, and the place where the data were aggregated, stored, and analyzed. The research ethics committee of Women’s Hospital of Nanjing Medical University approved the study, and the parents of the infants gave written informed consent for the prospective part of the study. The same diagnostic criteria were applied to all the NICUs.

### Study design

The study population comprised infants with birth weight < 1500 g and gestational age (GA) ≤34 weeks, hospitalized in the 19 NICUs in 2018, whose data were submitted to the multicenter clinical research database for breastfeeding quality improvement in Jiangsu province. Premature infants who began enteral feeding more than 2 weeks after birth and/or stayed in the hospital for less than 28 days and/or who had major congenital malformations or genetic metabolic diseases were excluded from the study.

In each hospital’s policy, all mothers were strongly encouraged to provide breast milk for their premature infants. Donor milk and preterm formula were available if own mother’s milk was insufficient. Intravenous nutrition was continued until a daily enteral intake of 150 mL/(kg · day) was reached. Human milk was fortified when human milk feeding reached 100 mL/(kg · day). Human milk included the infant’s own mother’ milk and donor milk. The effect of various doses of human milk on neonatal morbidity were compared. Human milk intake was classified according to a daily mean intake of 1–24 mL, 25–49 mL, or ≥ 50 mL/ (kg · day) to week 4 of life, and the groups were compared with a reference group receiving no human milk.

### Data collection

The database was developed for Improving Mother Milk Feeding Benefits in Neonatal Intensive Care Units (Clinicaltrials.gov#NCT03453502). This study entitled “The Dose-Dependent Effect of Human Milk on Bronchopulmonary Dysplasia in Very Low Birth Weight Infants” came from this database. The clinical data of eligible patients admitted to NICUs between January 1, 2018, and December 31, 2018, were collected these patients comprised the present study population. Neonatal data were collected including sex, birth weight, GA, small for GA, 5-min Apgar score, Score for Neonatal Acute Physiology with Perinatal Extension II (SNAPPE-II), and neonatal severity scores [9]. The use of mechanical ventilator (MV), time on MV, length of hospital stay, and time of full enteral feeding were also recorded. Neonatal outcomes examined included the incidences of BPD, necrotizing enterocolitis (NEC), late-onset sepsis (LOS), and extrauterine growth retardation (EUGR). Nutritional intake was recorded daily for 4 weeks after birth, including volume and type of enteral intake.

### Definitions

Full feeding was defined as full enteral feeding with no intravenous intake. BPD was defined as the need for supplementary oxygen for 28 days or more, and classified as mild, moderate, or severe BPD following the 2005 consensus [10]. LOS was diagnosed by the presence of clinical signs of sepsis and confirmed by blood culture after 3 days of life. NEC and severity grades of NEC were defined according to Bell’s stage [11]. EUGR was defined as body weight being lower than tenth percentile at 36 weeks’ postmenstrual age (PMA) or at the time of hospital discharge.

### Statistical analysis

Statistical analyses were performed using SPSS 22.0. Descriptive statistics included the mean and standard deviation for continuous variables following a normal distribution; median and interquartile range for skewed variables; and frequencies and percentages for categorical variables. We used The chi-square test, Kruskal-Wallis test, and one way analysis of variance were used to compare the varying dosages of human milk daily with neonatal data and clinical information.

Logistic regression analyses were performed to examine the associations between the volume of human milk daily and neonatal complications, with adjustment for potential confounders. The risk was reported as an odds ratio (OR) with 95% confidence interval (CI). Multivariate analysis was used to adjust for confounding variables including GA, small for GA (< 10 tenth percentile), sex, multiple births, cesarean section, 5-min Apgar score ≤ 7; SNAPPE-II; neonatal critical score, and mechanical ventilation time ≥ 7 days. A *P* value < 0.05 was considered statistically significant. In order to rule out multicollinearity between independent variables, the test of multicollinearity was performed. Major income was the dependent variable respectively. Gestational age, small for gestational age, multiple births, cesarean section, 5’Apgar score ≤ 7, neonatal critical score, Score for Neonatal Acute Physiology II and/or mechanical ventilation time ≥ 7 days were the independent variables. The variance inflation factors (VIF) as a diagnostic tool of multicollinearity was both less than 5 in our studies, which indicated there was no linear intercorrelation between explanatory variables.

## Results

**Fig. 1:**
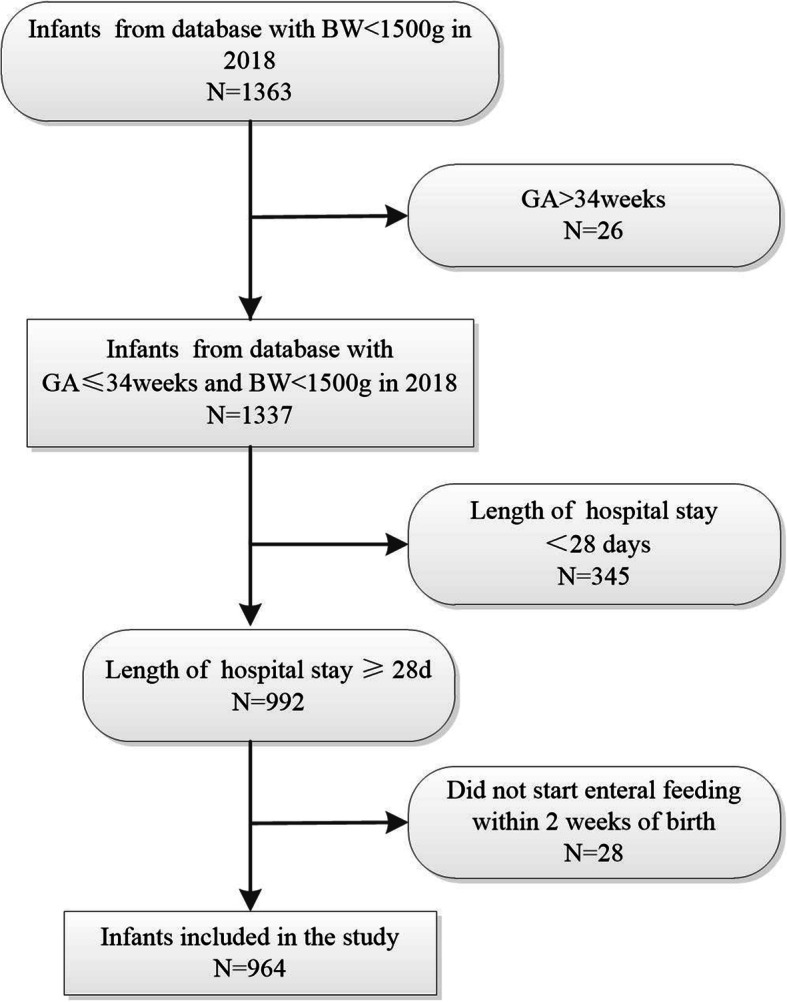
Flow diagram of the selection of the study population. BW, Birth weight; GA, gestational age

A total of 1363 VLBW infants were recruited from 19 hospitals during the time frame. Including 1337 infants with GA ≤34 weeks. Of these, 345 cases had often hospital stay of fewer than 28 days and 28 did not begin milk feeding within 2 weeks of life. These were all excluded, leaving 964 infants that fulfilled the inclusion criteria (Fig. 1).

The characteristics of the four groups by mean daily volume of human milk are shown in Table 1. A total of 279 (28.9%) of the 964 infants received exclusive preterm formula. A total of 853 (71.1%) received human milk, all of whom also received preterm formula as needed to achieve a full enteral intake. A total of 128 (13.3%) received a mean volume of 1–24 mL/(kg · day) of human milk daily during the first 4 weeks of life, 139 (14.4%) received 25–49 mL/(kg · day), and 418 (43.4%) received ≥50 mL/(kg · day). Statistically significant differences were found in GA, cesarean section, multiple births, small for GA, SNAPPE-II, and time on total enteral nutrition (TEN) among the four groups.

**Table 1:** Infant characteristics according to human milk intake in first 4 weeks of life

Characteristic	Daily volume of human milk, ml/kg body weight						
	0	1–24	25–49	≥50	sum	Statistical value	*P*-value
Number of subjects, n (%)	279 (28.9)	128 (13.3)	139 (14.4)	418 (43.4)	964		
Gender (male), n (%)	139 (49.8)	72 (56.3)	73 (52.5)	214 (51.2)	498 (51.7)	χ^2^ = 1.535	0.674
Birth weight (grams), mean ± SD	1215 ± 179	1184 ± 186	1228 ± 165	1219 ± 186	1215 ± 181	F = 1.594	0.189
1250–1499, n (%)	144 (51.6)	62 (48.4)	67 (48.2)	215 (51.4)	488 (50.6)		
1000–1249, n (%)	95 (34.1)	43 (33.6)	58 (41.7)	153 (36.6)	349 (36.2)		
750–999, n (%)	39 (14)	21 (16.4)	14 (10.1)	41 (9.8)	115 (11.9)		
< 750, n (%)	1 (0.4)	2 (1.6)	0 (0)	9 (2.2)	12 (1.2)		
Gestational age (weeks), mean ± SD	30.1 ± 1.8	29.6 ± 2.0	29.7 ± 1.8	29.5 ± 1.9	29.7 ± 1.9	F = 6.466	0.000
32–34, n (%)	50 (17.9)	21 (16.4)	18 (12.9)	50 (12.0)	139 (14.4)		
30–31, n (%)	104 (37.3)	28 (21.9)	40 (28.8)	106 (25.4)	278 (28.8)		
28–29, n (%)	96 (34.4)	54 (42.2)	63 (45.3)	190 (45.5)	403 (41.8)		
<28, n (%)	29 (10.4)	25 (19.5)	18 (12.9)	72 (17.2)	144 (14.9)		
Cesarean section, n (%)	175 (62.7)	63 (49.2)	70 (50.4)	219 (52.4)	525 (54.7)	χ^2^ = 10.820	0.013
Multiple births, n (%)	55 (19.7)	31 (24.2)	45 (32.4)	106 (25.5)	237 (24.6)	χ^2^ = 13.196	0.040
5’Apgar score ≤ 7, n (%)	97 (34.8)	38 (29.7)	28 (20.1)	65 (15.6)	228 (23.7)	χ^2^ = 37.812	0.000
Small for GA, n (%)	24 (8.6)	7 (5.5)	16 (11.5)	19 (4.5)	66 (6.8)	χ^2^ = 9.941	0.019
Neonatal critical score, mean ± SD	96 ± 7	96 ± 6	97 ± 6	97 ± 7	96 ± 7	F = 1.095	0.350
SNAPPE-II, median (P25, P75)	18 (5, 35)	15 (7, 31)	12 (5, 21)	9 (0, 21)	13 (5, 26)	Z = 40.598	0.000
MV, n (%)	90 (32.3)	55 (43.0)	53 (38.1)	172 (41.1)	370 (38.4)	χ^2^ = 6.919	0.075
Time on MV ≥7 days, n (%)	36 (12.9)	16 (12.5)	14 (10.1)	35 (8.4)	101 (10.5)	χ^2^ = 4.306	0.230
Time on TEN (days), median (P25, P75)	28 (21, 39)	30 (22, 41)	24 (16, 30)	19 (13, 26)	23 (16, 32)	Z = 149.286	0.000
Length of stay (days), median (P25, P75)	45 (37, 57)	47 (36, 58)	45 (37, 56)	43 (35, 53)	44 (36, 56)	Z = 6.246	0.100

Compared with infants receiving no human milk, those with the highest volume of human milk [≥50 mL/(kg · day)] had a lower incidence of preterm complications of BPD [27.5% for those receiving ≥50 mL/(kg · day) human milk daily vs 40.1% for those receiving 0 mL/(kg · day) human milk]; moderate and severe BPD [8.9% ≥50 mL/(kg · day) vs 16.1% 0 mL/(kg · day)]; NEC [3.8% 50 mL/(kg · day) vs 10.8% 0 mL/(kg · day)]; LOS [9.3% ≥50 mL/(kg · day) vs 19.7% mL/(kg · day)]; and EUGR [38.5% ≥50 mL/(kg · day) vs 57.6% 0 mL/(kg · day)]. No effect of 1–24 mL/(kg · day) or 25–49 mL/(kg · day) of human milk on BPD and moderate-to-severe BPD was observed daily during the first 4 weeks of life (Table 2).

**Table 2:** Neonatal outcomes of various doses of human milk intake in first 4 weeks of life

Neonatal outcomes	Daily volume of human milk, ml/kg body weight						
	0	1–24	25–49	≥50	sum	Statistical value	*P*-value
Main outcomes, n (%)							
BPD	112 (40.1)	52 (40.6)	48 (34.5)	115 (27.5)	327 (33.9)	χ^2^ = 15.069	0.002
Moderate-severe BPD	45 (16.1)	15 (11.7)	13 (9.4)	37 (8.9)	110 (11.4)	χ^2^ = 9.447	0.024
Secondary outcomes, n (%)							
NEC	30 (10.8)	18 (14.1)	22 (15.8)	16 (3.8)	86 (8.9)	χ^2^ = 26.821	0.000
NEC (≥Bell’s stage 2)	3 (1.1)	3 (2.3)	3 (2.2)	5 (1.2)	14 (1.5)	χ^2^ = 1.664	0.645
Late-onset sepsis	55 (19.7)	34 (26.6)	27 (19.4)	39 (9.3)	155 (16.1)	χ^2^ = 28.419	0.000
EUGR	160 (57.6)	69 (54.3)	60 (43.8)	155 (38.5)	444 (47.0)	χ^2^ = 27.531	0.000

*BPD* Bronchopulmonary dysplasia; *EUGR* extrauterine growth retardation; *GA* gestational age; *MV* mechanical ventilation; *TEN* total enteral nutrition; *NEC* necrotizing enterocolitis; *SD* standard deviation; *SNAPPE-II* Score for Neonatal Acute Physiology II

Compared with infants receiving no human milk, those receiving ≥50 mL/(kg · day) human milk had lower odds of BPD (adjusted OR [AOR] 0.453; 95% CI: 0.309, 0.666); moderate and severe BPD (AOR 0.430; 95% CI: 0.249, 0.742); NEC (AOR 0.314; 95% CI: 0.162, 0.607); LOS (AOR 0.420; 95% CI: 0.263, 0.673); and EUGR (AOR 0.685; 95% CI: 0.479, 0.979) after adjustment for confounders (Table 3).

**Table 3:** Logistic regression analyses examining protective effect on neonatal morbidity of various doses of human milk versus no human milk in first 4 weeks of life

Neonatal morbidity	Daily volume of human milk (ml/kg)	Univariate	*P*-value	Multivariate	*P*-value
BPD^a^	0	OR = 1		OR = 1	
	1–24	1.020 (0.666, 1.563)	0.927	0.811 (0.496, 1.325)	0.403
	25–49	0.786 (0.515, 1.201)	0.267	0.746 (0.459, 1.213)	0.237
	≥50	0.566 (0.410, 0.781)	0.001	0.453 (0.309, 0.666)	0.000
Moderate-severe BPD^a^	0	OR = 1		OR = 1	
	1–24	0.690 (0.369, 1.291)	0.246	0.501 (0.246,1.013)	0.054
	25–49	0.537 (0.279, 1.032)	0.062	0.549 (0.267,1.129)	0.103
	≥50	0.505 (0.317, 0.803)	0.004	0.430 (0.249,0.742)	0.002
NEC^b^	0	OR = 1		OR = 1	
	1–24	1.358 (0.726, 2.540)	0.338	1.208 (0.626,2.331)	0.574
	25–49	1.561 (0.863, 2.822)	0.141	1.631 (0.870,3.059)	0.127
	≥50	0.330 (0.176, 0.618)	0.001	0.314 (0.162.0.607)	0.001
NEC(≥Bell’s stage 2)^b^	0	OR = 1		OR = 1	
	1–24	2.208 (0.440, 11.093)	0.336	1.244 (0.198, 7.823)	0.816
	25–49	2.029 (0.404, 10.188)	0.390	2.037 (0.387, 10.714)	0.401
	≥50	1.114 (0.264, 4.698)	0.883	0.854 (0.193, 3.786)	0.836
Later onset sepsis^b^	0	OR = 1		OR = 1	
	1–24	1.473 (0.902, 2.406)	0.122	1.413 (0.851, 2.346)	0.182
	25–49	0.982 (0.588, 1.641)	0.944	1.038 (0.607, 1.774)	0.892
	≥50	0.419 (0.269, 0.652)	0.000	0.420 (0.263, 0.673)	0.000
EUGR^a^	0	OR = 1		OR = 1	
	1–24	0.877 (0.575, 1.339)	0.544	1.287 (0.803,2.062)	0.294
	25–49	0.575 (0.380, 0.868)	0.009	0.701 (0.434.1.132)	0.147
	≥50	0.461 (0.338, 0.629)	0.000	0.685 (0.479,0.979)	0.038

*BPD* Bronchopulmonary dysplasia; *CI* confidence interval; *EUGR* extrauterine growth retardation; *NEC* necrotizing enterocolitis; *OR* odds ratio

^a^Adjusted for gestational age, small for gestational age, multiple births, cesarean section, 5’Apgar score ≤ 7, neonatal critical score, Score for Neonatal Acute Physiology II; mechanical ventilation time ≥ 7 days

^b^Adjusted for gestational age, small for gestational age, multiple births, cesarean section, 5’Apgar score ≤ 7; Score for Neonatal Acute Physiology II, neonatal critical score

Exclusive human milk feeding infants were divided into two subgroups with ≥50 mL/(kg · day) donor milk and ≥ 50 mL/(kg · day) own mother’s milk to clarify further whether donor milk had the same protective effect on BPD and other complications of preterm infants as own mother’s milk. The effect on BPD and other complications was compared between the two groups. The results showed no statistically significant difference in BPD, moderate-to-severe BPD, NEC, LOS, and EUGR (Table 4).

**Table 4:** The effect on neonatal morbidity of donor milk versus maternal milk in first 4 weeks of life

Subgroup	BPD n(%)	Moderate-severe BPD n(%)	NEC n(%)	NEC(≥Bell’s stage 2 n(%))	Later onset sepsis n(%)	EUGR n(%)
Donor milk ≥50 ml/kg/d	21 (14.3)	21 (0)	21 (0)	21 (0)	21 (0)	21 (36.8)
Maternal milk ≥50 ml/kg/d	127 (26.0)	127 (9.4)	127 (4.7)	127 (0.8)	127 (11.8)	127 (30.2)
Statistical value	1.340	2.159	1.034	0.166	2.760	0.345
*P*-value	0.247	0.142	0.309	0.683	0.097	0.557

## Discussion

Until recently, data suggesting a beneficial impact of human milk feeding on BPD were limited. Some studies concluded that human milk decreased the incidence of BPD [12–15] the result of other studies were contradictory [16, 17]. The present study found that human milk might reduce the incidence of BPD, with a dose-dependent relationship between human milk and the occurrence of BPD.

The proportion of preterm infants receiving breastfeeding in NICUs increased from 23.0% in 2005 to 37.2% in 2015 in China [8, 18] with an improved understanding of breastfeeding and strategies to promote breastfeeding The breastfeeding rates increased in NICUs of China. The proportion of exclusively formula-fed VLBW infants with a hospital stay of ≥28 days in this study was 28.9%, less than one-third of the total. The proportion of mixed feeding of human milk and formula was significantly higher than that of exclusive breastfeeding and exclusive formula – feeding due to limitations from various factors. This led to the question regarding the dose-dependent effect of human milk on the risk of BPD and other morbidities in VLBW infants. Several meta-analyses [12, 15, 19, 20] comparing human milk or own mother’s milk and any human milk with exclusive formula drew different conclusions, although most found that exclusive human milk feeding was associated with decreased incidence of BPD. Partially receiving human milk was also shown to have a protective effect compared with exclusive formula feeding, but the level of evidence was not high [12]. Patel et al. [21] revealed a 9.5% reduction in the odds of BPD for each 10% increase in enteral feedings consisting of mothers’ milk received from birth to 36 weeks PMA. Another study found that the risk of BPD reduced when the average breast milk volume given was more than 7 mL/(kg · day) at 42 days after birth [22]. This dose of human milk was far lower than that in the present study. The difference might be explained by different time period and different GA. The GA in the aforementioned study was less than 32 weeks and the time of feeding was 42 days after birth. However, while the gestational age in the present study was less than and equal to 34 weeks and the time of feeding was 28 days after birth. The time of feeding in the present study was selected according to the definition of BPD.

Furman et al. [17] and Schanler et al. [16, 23] found that feeding with at least 50 mL/(kg · day) of human milk reduced the incidence of LOS. However, the effect on NEC and BPD was not consistent, due to a limited sample size. The detailed clinical and feeding information of VLBW infants from 19 NICUs in Jiangsu province in 2018 was collected, providing a sufficiently large sample. At least 50 mL/(kg · day) of human milk daily given up to the end of the fourth week of life decreased the rates of BPD, as well as NEC, LOS, and EUGR in VLBW infants.

Infection is a risk factor for BPD, which alters lung development through inflammatory cytokines. Bioactive components of human milk can contribute to the development of the immunity system in preterm infants and reduce the chance of infection. Human milk can reduce the occurrence of BPD by reducing the incidence of sepsis and NEC.

Oxidative stress is a common pathway shared by BPD, NEC, sepsis, and EUGR, it causes lipid, protein, and DNA damage. Preterm infants have poor antioxidant defenses in response to oxidative challenge, because the physiologic increase in antioxidant ability occurs at the end of term birth [24–28]. Therefore, preterm infants are more susceptible to reactive oxygen species (ROS)-induced damage. Inadequate nutrition increases oxidative stress [28]. Human milk has many bioactive components that prevent oxidative stress [7, 29, 30]. The composition of human milk can vary with the infant’s requirements according to its age and other characteristics [31, 32]. High-dose human milk feeding may provide nutritional and bioactive components that mitigate oxidative stress, inflammation, and dietary inadequacies [33, 34]. Furthermore, these protective components of human milk are highly concentrated as the volume of human milk increases.

The human milk included own mother’s milk and donor milk. Only two of the NICUs in present multicenter study. The volumes of donor milk were low and were combined with human milk. The methods of storage and disinfection of donor milk may also have affected its nutritional composition [35, 36]. A subgroup analysis was performed to verify dose-related effects of donor milk and own mother’s milk. The donor milk intake of ≥50 mL/(kg · day) during the first 4 weeks of life also reduced the incidence of BPD, NEC, LOS, and EUGR as own mother’s milk.

A limitation of the present study is was baseline differences in the participants who received different volumes of human milk daily for the first 4 weeks of life. The statistical analyses adjusted for these differences; however, it was possible that not all the differences between these groups could be controlled statistically. Additionally, very few infants were fed donor milk. The sample size needs to be enlarged for further verification.

## Conclusion

A daily threshold amount of at least 50 mL/(kg · day) human milk throughout the first 4 weeks of life reduced the risk of BPD as well as NEC, LOS, and EUGR in VLBW infants.

## Supplementary information

**Additional file 1.** Multicollinearity Test.

## Data Availability

The datasets used and/or analyzed during the current study are available from corresponding author on reasonable request.

## Abbreviations

AOR: Adjusted odds ratio (AOR)
BPD: Bronchopulmonary dysplasia
CI: Confidence interval
EUGR: extrauterine growth retardation
GA: Gestational age
LOS: Late-onset sepsis
MV: Mechanical ventilator
NEC: Necrotizing enterocolitis
PMA: Postmenstrual age
ROS: Reactive oxygen species
SNAPPE-II: Score for Neonatal Acute Physiology with Perinatal Extension II
TEN: Total enteral nutrition
VLBW: Very low birth weight
