# Supplementary material for: Dose-dependent effect of human milk on Bronchopulmonary dysplasia in very low birth weight infants
Source: BMC Pediatr. 2020 Nov 16;20:522. doi: 10.1186/s12887-020-02394-1 (PMC7666971; doi:10.1186/s12887-020-02394-1)
Supplement: Supplementary file 1 — Additional file 1. Multicollinearity Test. [file 12887_2020_2394_MOESM1_ESM.docx]

**Multicollinearity Test**

| **Coefficients ^a^** | | | | | | | | |
| --- | --- | --- | --- | --- | --- | --- | --- | --- |
| Model | | Unstandardized coefficients | | Standardized coefficients | T | Sig.  Tolerance | Collinear statistics | |
|  |  | B | Std. Error | Beta |  |  | Tolerance | VIF |
| 1 | （Constant） | 1.117 | .264 |  | 4.234 | .000 |  |  |
|  | Gestational age | -.010 | .008 | -.042 | -1.299 | .194 | .981 | 1.019 |
|  | SGA | -.161 | .068 | -.082 | -2.381 | .017 | .864 | 1.158 |
|  | Cesarean section | -.136 | .032 | -.144 | -4.324 | .000 | .937 | 1.067 |
|  | SNAPPE-II | .003 | .002 | .088 | 1.793 | .073 | .433 | 2.310 |
|  | Neonatal critical score | -.005 | .002 | -.065 | -1.927 | .054 | .904 | 1.106 |
|  | Daily volume of human milk intake in first 4 weeks of life | -.001 | .000 | -.104 | -3.148 | .002 | .945 | 1.058 |
|  | Multiple births | -.055 | .033 | -.054 | -1.668 | .096 | .993 | 1.007 |
|  | 5’Apgar score <7 | -.050 | .052 | -.045 | -.965 | .335 | .483 | 2.069 |
|  | Time on MV ≥7 days | .342 | .055 | .211 | 6.203 | .000 | .898 | 1.114 |
| a. [Dependent](javascript:;) [variable](javascript:;): BPD | | | | | | | | |

| **Coefficients ^a^** | | | | | | | | |
| --- | --- | --- | --- | --- | --- | --- | --- | --- |
| Model | | Unstandardized coefficients | | Standardized coefficients | T | Sig. | Collinear statistics | |
|  |  | B | Std. Error | Beta |  |  | Tolerance | VIF |
| 1 | （Constant） | .031 | .169 |  | .183 | .855 |  |  |
|  | Gestational age | .003 | .005 | .024 | .692 | .489 | .984 | 1.016 |
|  | SGA | -.066 | .043 | -.056 | -1.521 | .129 | .871 | 1.148 |
|  | Cesarean section | -.008 | .020 | -.014 | -.400 | .689 | .919 | 1.088 |
|  | Multiple births | -.017 | .021 | -.027 | -.784 | .433 | .995 | 1.005 |
|  | 5’Apgar score <7 | .083 | .034 | .122 | 2.448 | .015 | .478 | 2.090 |
|  | Neonatal critical score | .000 | .002 | -.004 | -.112 | .911 | .908 | 1.101 |
|  | SNAPPE-II | .003 | .001 | .179 | 3.501 | .000 | .451 | 2.216 |
|  | Daily volume of human milk intake in first 4 weeks of life | -.001 | .000 | -.082 | -2.323 | .020 | .939 | 1.065 |
| a. [Dependent](javascript:;) [variable](javascript:;): NEC | | | | | | | | |

| **Coefficients ^a^** | | | | | | | | | |
| --- | --- | --- | --- | --- | --- | --- | --- | --- | --- |
| Model | | Unstandardized coefficients | | Standardized coefficients | T | Sig. | Collinear statistics | |  |
|  |  | B | Std. Error | Beta |  |  | Tolerance | VIF |  |
| 1 | （Constant） | .011 | .071 |  | .151 | .880 |  |  |  |
|  | Gestational age | .001 | .002 | .016 | .452 | .651 | .984 | 1.016 |  |
|  | SGA | -.020 | .018 | -.041 | -1.116 | .265 | .871 | 1.148 |  |
|  | Cesarean section | .005 | .009 | .020 | .556 | .578 | .919 | 1.088 |  |
|  | Multiple births | -.019 | .009 | -.072 | -2.066 | .039 | .995 | 1.005 |  |
|  | 5’Apgar score <7 | .023 | .014 | .081 | 1.612 | .107 | .478 | 2.090 |  |
|  | Neonatal critical score | -8.225E-7 | .001 | .000 | -.001 | .999 | .908 | 1.101 |  |
|  | SNAPPE-II | .000 | .000 | .029 | .556 | .578 | .451 | 2.216 |  |
|  | Daily volume of human milk intake in first 4 weeks of life | .000 | .000 | -.041 | -1.135 | .257 | .939 | 1.065 |  |
| a. [Dependent](javascript:;) [variable](javascript:;): NEC (≥Bell’s stage 2) | | | | | | | | | |

| **Coefficients ^a^** | | | | | | | | |
| --- | --- | --- | --- | --- | --- | --- | --- | --- |
| Model | | Unstandardized coefficients | | Standardized coefficients | T | Sig. | Collinear statistics | |
|  |  | B | Std. Error | Beta |  |  | Tolerance | VIF |
| 1 | （Constant） | .404 | .216 |  | 1.871 | .062 |  |  |
|  | Gestational age | .006 | .006 | .031 | .889 | .374 | .984 | 1.016 |
|  | SGA | .029 | .056 | .019 | .523 | .601 | .871 | 1.148 |
|  | Cesarean section | -.021 | .026 | -.028 | -.787 | .432 | .919 | 1.088 |
|  | Multiple births | -.038 | .027 | -.049 | -1.413 | .158 | .995 | 1.005 |
|  | 5’Apgar score <7 | .004 | .043 | .005 | .091 | .927 | .478 | 2.090 |
|  | Neonatal critical score | -.002 | .002 | -.040 | -1.120 | .263 | .908 | 1.101 |
|  | SNAPPE-II | .002 | .001 | .103 | 2.015 | .044 | .451 | 2.216 |
|  | Daily volume of human milk intake in first 4 weeks of life | -.001 | .000 | -.068 | -1.901 | .058 | .939 | 1.065 |
| a. [Dependent](javascript:;) [variable](javascript:;): LOS | | | | | | | | |

| **Coefficients ^a^** | | | | | | | | |
| --- | --- | --- | --- | --- | --- | --- | --- | --- |
| Model | | Unstandardized coefficients | | Standardized coefficients | T | Sig. | Collinear statistics | |
|  |  | B | Std. Error | Beta |  |  | Tolerance | VIF |
| 1 | （Constant） | .205 | .175 |  | 1.176 | .240 |  |  |
|  | Gestational age | -.006 | .005 | -.037 | -1.114 | .265 | .983 | 1.018 |
|  | SGA | -.067 | .045 | -.052 | -1.484 | .138 | .864 | 1.157 |
|  | Cesarean section | -.075 | .021 | -.121 | -3.557 | .000 | .915 | 1.092 |
|  | Multiple births | -.052 | .022 | -.078 | -2.387 | .017 | .993 | 1.007 |
|  | 5’Apgar score <7 | -.014 | .035 | -.019 | -.402 | .687 | .478 | 2.091 |
|  | Neonatal critical score | .001 | .002 | .014 | .404 | .686 | .901 | 1.110 |
|  | SNAPPE-II | .003 | .001 | .149 | 3.037 | .002 | .437 | 2.290 |
|  | Daily volume of human milk intake in first 4 weeks of life | .000 | .000 | -.064 | -1.898 | .058 | .937 | 1.067 |
|  | Time on MV ≥7 days | .232 | .037 | .218 | 6.347 | .000 | .897 | 1.115 |
| a. [Dependent](javascript:;) [variable](javascript:;): moderate-severe BPD | | | | | | | | |

| **Coefficients ^a^** | | | | | | | | |
| --- | --- | --- | --- | --- | --- | --- | --- | --- |
| Model | | Unstandardized coefficients | | Standardized coefficients | T | Sig. | Collinear statistics | |
|  |  | B | Std. Error | Beta |  |  | Tolerance | VIF |
| 1 | （Constant） | .531 | .283 |  | 1.881 | .060 |  |  |
|  | Gestational age | .003 | .008 | .012 | .348 | .728 | .982 | 1.018 |
|  | SGA | .438 | .073 | .213 | 6.017 | .000 | .863 | 1.159 |
|  | Cesarean section | .207 | .034 | .207 | 6.024 | .000 | .916 | 1.092 |
|  | Multiple births | -.030 | .036 | -.028 | -.843 | .400 | .993 | 1.007 |
|  | 5’Apgar score <7 | .049 | .056 | .041 | .863 | .388 | .474 | 2.108 |
|  | Neonatal critical score | -.005 | .003 | -.067 | -1.936 | .053 | .898 | 1.114 |
|  | SNAPPE-II | .003 | .002 | .086 | 1.723 | .085 | .434 | 2.306 |
|  | Daily volume of human milk intake in first 4 weeks of life | -7.888E-5 | .000 | -.007 | -.198 | .843 | .938 | 1.066 |
|  | Time on MV ≥7 days | .113 | .059 | .066 | 1.906 | .057 | .898 | 1.114 |
| a. [Dependent](javascript:;) [variable](javascript:;): EUGR | | | | | | | | |
